# Four new mitochondrial genomes for the basal bee family Melittidae (Hymenoptera: Apoidea)

**DOI:** 10.1080/23802359.2020.1871435

**Published:** 2021-02-09

**Authors:** Jana Nickel, Carla Violetta Reinbold, Michael Kuhlmann, Mathilde Cordellier, Martin Husemann

**Affiliations:** a Institute for Zoology, Universität Hamburg, Hamburg, Germany; b Center for Bioinformatics, University of Hamburg, Hamburg, Germany; c Centrum für Naturkunde, Universität Hamburg, Hamburg, Germany; d Zoological Museum, University of Kiel, Kiel, Germany

**Keywords:** Illumina sequencing, Melittidae, Anthophila, *Dasypoda*, *Melitta*

## Abstract

We present four new complete mitochondrial genomes for *Dasypoda hirtipes, Melitta schultzei*, *Capicola nanula* and *Samba griseonigra* belonging to the basally branching bee family Melittidae covering four genera in three tribes (Melittini, Hesperaspini, Dasypodaini) and two subfamilies (Melittinae, Dasypodainae). The mitogenomes vary between 15,884 and 20,324 bp in length and consist of the typical set of 13 protein-coding genes, 22 tRNAs, two rRNAs and the control region. These new mitogenomes raise the number of available mitochondrial genomes for the family Melittidae to five and will help to shed light on the phylogenetic relationships within Melittidae and their position within the Anthophila.

## Introduction

The family Melittidae includes more than 200 solitary bee species and occurs in temperate and xeric ecosystems of the Nearctic and the Old World (Michez et al. 2009). It has been suggested that Melittidae is a monophyletic group and basal to all other Anthophila (Peters et al. 2017), but multi-gene and mitogenomic data suggest paraphyletic Melittidae and point to a close relationship with Colletidae (Danforth et al. 2006; Kahnt et al. 2015). However, so far only a single mitogenome of the Melittidae is available (*Rediviva intermixta* (Cockerell, 1934); Kahnt et al. 2015). Therefore, in this study, we newly sequenced and assembled the mitochondrial genomes of four species belonging to the family Melittidae representing four different genera in three tribes and two subfamilies: *Dasypoda hirtipes* Fabricius, 1793, *Melitta schultzei* Friese, 1909, *Capicola nanula* Cockerell, 1936 and *Samba griseonigra* (Michener, 1981). This new data will allow a more comprehensive assessment of the evolution of mitogenomes in this putatively basal bee family in the future.

## Materials and methods

Material of *D. hirtipes* was collected during a field trip to Pevestorf (Lower Saxony, Germany); *Melitta schultzei*, *C. nanula* and *S. griseonigra* were collected in South Africa by MK (Supplemental Table 1). Specimens were conserved pinned and dried, or preserved in ethanol. After collection, specimens were identified morphologically by MK using the keys provided by Eardley and Kuhlmann (2006), and Michez et al. (2007, 2010). Remaining parts of specimens and labels are deposited in the Zoological Museum Hamburg under the accessions (ZMH 841435-841438). DNA voucher numbers match specimen accessions. The last author (MH) is the responsible curator of specimens and DNA vouchers.

Genomic DNA was extracted from *D*. *hirtipes* using salt-extraction (Aljanabi and Martinez, 1997) and libraries were prepared using NEB Next® Ultra™ II DNA Library Prep Kit for Illumina^®^ (New England Biolabs, Ipswich, MA, USA). Genomic DNA was extracted from *M*. *schultzei*, *C*. *nanula* and *S*. *griseonigra* using the EchoLUTION Blood DNA HiYield Kit (BioEcho, Dormagen, Germany). Bee vouchers were used up completely; DNA samples are stored in the biobank of the CeNak. Libraries were prepared using the Nextera DNA Flex Library Prep Kit (Illumina, San Diego, CA, USA). Paired-end and single-end reads were sequenced on the Illumina MiSeq platform (Table S1). Adapter trimming and quality filtering for all reads was performed using Trimmomatic v. 0.38 (Bolger et al. 2014). The resulting trimmed reads were used to produce mitochondrial genome assemblies using the ‘de novo assembly’ and ‘find mitochondrial scaffold’ modules provided in MitoZ v. 2.4 with default settings (Meng et al. 2019). For *D*. *hirtipes* and *M*. *schultzei*, this was not sufficient to recover a complete mitogenome and the multi-kmer mode in MitoZ was used with a kmer length of 99 and 31, respectively, to achieve the longest continuous mitochondrial contig. The preliminary mitogenomes (∼13,000 bp) were then used as the reference genomes for mitochondrial baiting and iterative mapping as implemented in MITObim v. 1.9.1 (Hahn et al. 2013). Complete mitochondrial sequences were annotated using the MITOS2 webserver (Bernt et al. 2013).

## Results and discussion

The complete mitogenomes of *D*. *hirtipes*, *M***.** *schultzei*, *C*. *nanula*, and *S*. *griseonigra* are 18,594 bp, 20,324 bp, 15,884 bp, and 16,978 bp in length, respectively and all consist of 13 protein-coding genes (PCGs), 22 tRNA genes, 2 rRNA genes and the non-coding mitochondrial control region (Supplemental Table 1). The average A + T content of the mitogenomes ranged from 74.15% (*D*. *hirtipes*) to 84.97% (*S*. *griseonigra*). The overall gene content and base composition for all species was similar to *Rediviva intermixta* (79.9% A + T content) and Colletidae species (Kahnt et al. 2015). Four protein-coding genes (PCGs) were encoded on the plus strand in *C*. *nanula* and *S*. *griseonigra*, 8 PCGs in *D*. *hirtipes* and 9 PCGs in *M***.** *schultzei* with the remaining PCGs on the minus strand. Depending on the species, five to six PCGs used ATT as the start codon, between four and six used ATG and between one and three used ATA. Most PCGs used the typical stop codon TAA, while in *D*. *hirtipes* one PCG terminated with TAG, in *S*. *griseonigra* three PCGs terminated with TAG and one PCG in *M***.** *schultzei, C*. *nanula*, and *S*. *griseonigra* ended with the incomplete termination signal T. The 12S rRNA and 16S rRNA of *D*. *hirtipes* which could be annotated completely were 753 bp and 1328 bp, respectively. The 22 tRNA genes range in size from 61 to 78 bp. The tRNA of *trnG* could not be recovered in *D*. *hirtipes*. The control region was split in all mitogenomes; similarly, *rrnL* was split in *S*. *griseonigra*, both *rrnL* and *rrnS* were split in *C*. *nanula* and *rrnS*, *cox3* and *atp6* were split in *M***.** *schultzei*. While the gene order of PCGs and rRNAs seems to be conserved among Anthophila, rearrangements of tRNA were found within the Melittidae which is common in Hymenoptera mitogenomes (Dowton et al. 2009). A phylogenetic assessment using the new mitochondrial genomes in a dataset together with all complete published mitochondrial genomes of Anthophila supports the monophyly of Melittidae and supports their basal position (Husemann et al. 2020).

## Data Availability

The genome sequence data that support the findings of this study are openly available in GenBank of NCBI at (https://www.ncbi.nlm.nih.gov/) under the accession no. MT985325-MT985328. All short read data is provided in ENA under accession number PRJEB41994.
